# Impact of Plasma p‐tau181 on Cognition, Motor Phenotypes, and Disease Course in ALS

**DOI:** 10.1002/acn3.70423

**Published:** 2026-05-04

**Authors:** Elisabeth Kasper, Annaliis Lehto, Nina Nordmann, Oliver Peters, Julian Hellmann, Josef Priller, Eike Jakob Spruth, Gabor C. Petzold, Ina Vogt, Patrick Weydt, Sarah Bernsen, Elisabeth Dinter, Björn Falkenburger, René Günther, Emrah Düzel, Wenzel Glanz, Matthis Synofzik, Lukas Beichert, Annika Spottke, Michael Wagner, Frederic Brosseron, Matthias C. Schmid, Anja Schneider, Stefan Teipel, Johannes Prudlo, Andreas Hermann

**Affiliations:** ^1^ Department of Neurology Rostock University Medical Center Rostock Germany; ^2^ German Center for Neurodegenerative Diseases (DZNE) Rostock/Greifswald Germany; ^3^ Translational Neurodegeneration Section “Albrecht Kossel”, Department of Neurology Rostock University Medical Center Rostock Germany; ^4^ German Center for Neurodegenerative Diseases (DZNE) Berlin Germany; ^5^ Charité—Universitätsmedizin Berlin, Corporate Member of Freie Universität Berlin and Humboldt‐Universität zu Berlin‐Institute of Psychiatry and Psychotherapy Berlin Germany; ^6^ Department of Psychiatry and Neurosciences Charité Universitätsmedizin Berlin Berlin Germany; ^7^ ECRC Experimental and Clinical Research Center Charité—Universitätsmedizin Berlin Berlin Germany; ^8^ Neuropsychiatry and Laboratory of Molecular Psychiatry, Department of Psychiatry and Psychotherapy Charité—Universitätsmedizin Berlin Berlin Germany; ^9^ Department of Psychiatry and Psychotherapy, School of Medicine and Health Technical University of Munich, and German Center for Mental Health (DZPG) Munich Germany; ^10^ University of Edinburgh and UK DRI Edinburgh UK; ^11^ German Center for Neurodegenerative Diseases (DZNE) Bonn Germany; ^12^ Department of Vascular Neurology University Hospital Bonn Bonn Germany; ^13^ Department of Neurology University of Bonn Bonn Germany; ^14^ German Center for Neurodegenerative Diseases (DZNE) Dresden Germany; ^15^ Department of Neurology University Hospital Carl Gustav Carus, Technische Universität Dresden Dresden Germany; ^16^ German Center for Neurodegenerative Diseases (DZNE) Magdeburg Germany; ^17^ Institute of Cognitive Neurology and Dementia Research Otto‐von‐Guericke University Magdeburg Germany; ^18^ Institute of Cognitive Neuroscience University College London London UK; ^19^ German Center for Neurodegenerative Diseases (DZNE) Tübingen Germany; ^20^ Division of Translational Genomics of Neurodegenerative Diseases Hertie Institute for Clinical Brain Research and Center of Neurology, University of Tübingen Tübingen Germany; ^21^ Department for Cognitive Disorders and Old Age Psychiatry University Hospital Bonn Bonn Germany; ^22^ Institute for Medical Biometry, Informatics and Epidemiology University Hospital Bonn Bonn Germany; ^23^ Department of Psychosomatic Medicine Rostock University Medical Center Rostock Germany

**Keywords:** Alzheimer disease, amyotrophic lateral sclerosis, plasma p‐tau181

## Abstract

Phosphorylated tau181 (p‐tau181), an Alzheimer's disease biomarker, was recently evaluated in amyotrophic lateral sclerosis (ALS). We investigated plasma p‐tau181 in 202 ALS/ALS‐FTD patients and 94 healthy controls, assessing cognitive performance, motor function, and longitudinal dynamics. Plasma p‐tau181 and NfL were significantly elevated in ALS, with p‐tau181 increasing over 1 year while NfL remained stable. Neither marker correlated with cognitive performance, and only NfL was associated with disease severity and progression. Plasma p‐tau181 was higher in patients with predominant lower motor neuron involvement. The results indicate that p‐tau181 reflects peripheral processes in ALS, providing a complementary, mechanistically distinct biomarker from NfL.

## 1 Introduction

While phosphorylated tau181 (p‐tau181) is an FDA‐ and EMA approved blood diagnostic biomarker for Alzheimer's disease (AD) [1], it has recently gained attention in amyotrophic lateral sclerosis (ALS). A number of studies reported elevated serum p‐tau181 in people living with ALS (plwALS), with comparable concentrations between ALS and AD [2, 3, 4]. Further, plasma p‐tau181 has been linked to lower motor neuron dysfunction [4]. Of interest, however, no correlation between p‐tau181 in CSF and serum levels was observed in plwALS, arguing against serum p‐tau181 originating solely from the central nervous system in case of ALS, and thus also against just being a marker of AD co‐pathology [2]. For comparison, neurofilament light chain (NfL), an established biomarker of axonal neurodegeneration in ALS, has consistently been reported to be markedly elevated people living with ALS [5].

Cognitive impairment is increasingly recognized as a key non‐motor feature in ALS, affecting up to 50% of patients. Deficits typically involve verbal fluency, executive function, language, and social cognition [6], but may also include memory dysfunction [7], a hallmark of AD.

In this study, we sought to extend previous findings on plasma p‐tau181 in ALS by examining its relationship with cognitive function and potential dissociation from the robust association with motor impairment. We hypothesized, that if plasma p‐tau181 primarily reflects peripheral processes, associations with cognitive performance—potentially indicative of AD co‐pathology—would not be expected. We further investigated longitudinal dynamics of plasma p‐tau181 in relation to NfL, and clinical parameters not yet systematically explored.

## 2 Methods

We analyzed 202 plwALS or with comorbid frontotemporal dementia (ALS‐FTD) as well as 94 healthy controls from two multicenter cohorts of the German Center for Neurodegenerative Diseases/DZNE (see supplement). All participants underwent standardized clinical assessment, including ALSFRS‐R [8] and were diagnosed according to the revised El Escorial Criteria [9] and categorized according to the King stages [10]. APOE genotype was dichotomized into ε4 carriers (ε3/ε4, ε4/ε4) and non‐carriers (ε2/ε4, ε2/ε3, ε3/ε3).

Key biomarkers analyzed included the neurodegeneration panel from CSF by manual method (for description see Supporting Information S1). ATN status was determined according to the ATN framework (amyloid deposition [A], tau pathology [T], and neurodegeneration [N]) using CSF Aβ42/40 ratio, p‐tau181, and total tau [1] applying our assay‐specific cut‐off values (see supplement) (0 = normal, 1 = early amyloid pathology, 2 = AD pathology, 3 = AD with neurodegeneration). Blood plasma measures of p‐tau181 were conducted using the Quanterix SIMOA Assay accordi ng to the manufacturer's instructions, with samples run in technical duplicates and a maximum accepted coefficient of variance of 20%. Additional to manufacturer's kit controls, an internal aliquoted plasma samples served as inter‐run control. NfL mean concentrations were determined using the SIMOA NF‐light Advantage kit on a HDI analyzer Quanterix by a blinded experimenter according to the manufacturer's instructions as previously described [11].

The neuropsychological assessment included the German version of the “Edinburgh Cognitive and Behavioural ALS Screen” (ECAS) [12, 13]. The ECAS comprises 15 subtests assessing five cognitive domains: ALS specific functions (verbal fluency, executive functions including social cognition, and language) and ALS‐non‐specific functions (memory and visuospatial abilities). We considered sub scores for each cognitive subdomain, as well as the ALS‐specific score, the ALS‐nonspecific score, and the ECAS total score. Cognitive impairments were classified according to the revised Strong criteria [14]. Based on this classification, all individuals with ALS without FTD were categorized as either “non‐impaired” (ALSni) or “cognitively impaired” (ALSci). Possible behavioral abnormalities and the corresponding classifications (ALSbi and ALScbi) were not included, as the present analysis focused specifically on cognitive domains. Patients with ALS and additional FTD were categorized according to Rascovsky et al. [15] and Gorno‐Tempini et al. [16], respectively.

For statistical analyses a linear mixed‐effects model approach was applied to both cross‐sectional and longitudinal data. Non‐parametric group statistics were used where appropriate. Associations between cognitive scores and biomarkers were analyzed using partial correlations controlling for age and corrected for multiple comparisons.

For further methodological details, please refer to the Supporting Information S1.

## 3 Results

**FIGURE 1:**
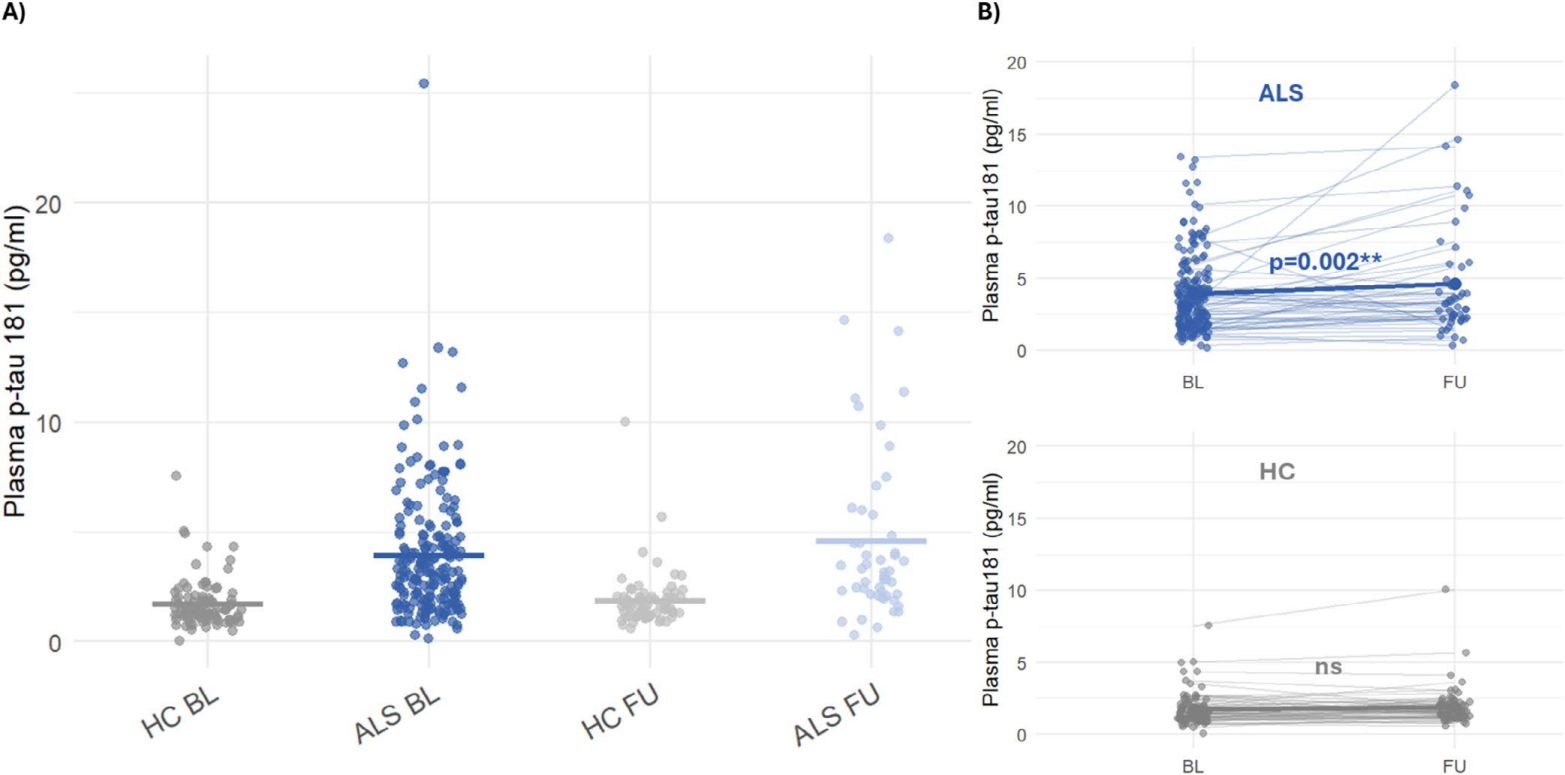
Baseline differences and longitudinal dynamics of plasma p‐tau181. (A) Group comparisons of plasma p‐tau181 at baseline and follow‐up in patients and healthy controls. Points represent individual values; horizontal bars indicate group means. (B) Longitudinal trajectories of plasma p‐tau181 in patients (top) and healthy controls (bottom). Lines connect repeated measures for individuals with data at both time points; points represent all available observations. BL, baseline; HC, healthy controls; FU, follow‐up (dark blue/dark gray = BL; light blue/light gray = FU). Significance levels from linear mixed‐effects models adjusted for age: *p < 0.05, **p < 0.01, ***p < 0.001. Linear mixed‐effects models were fitted using all available data, whereas longitudinal trajectories include only individuals with measurements at both time points.

**FIGURE 2:**
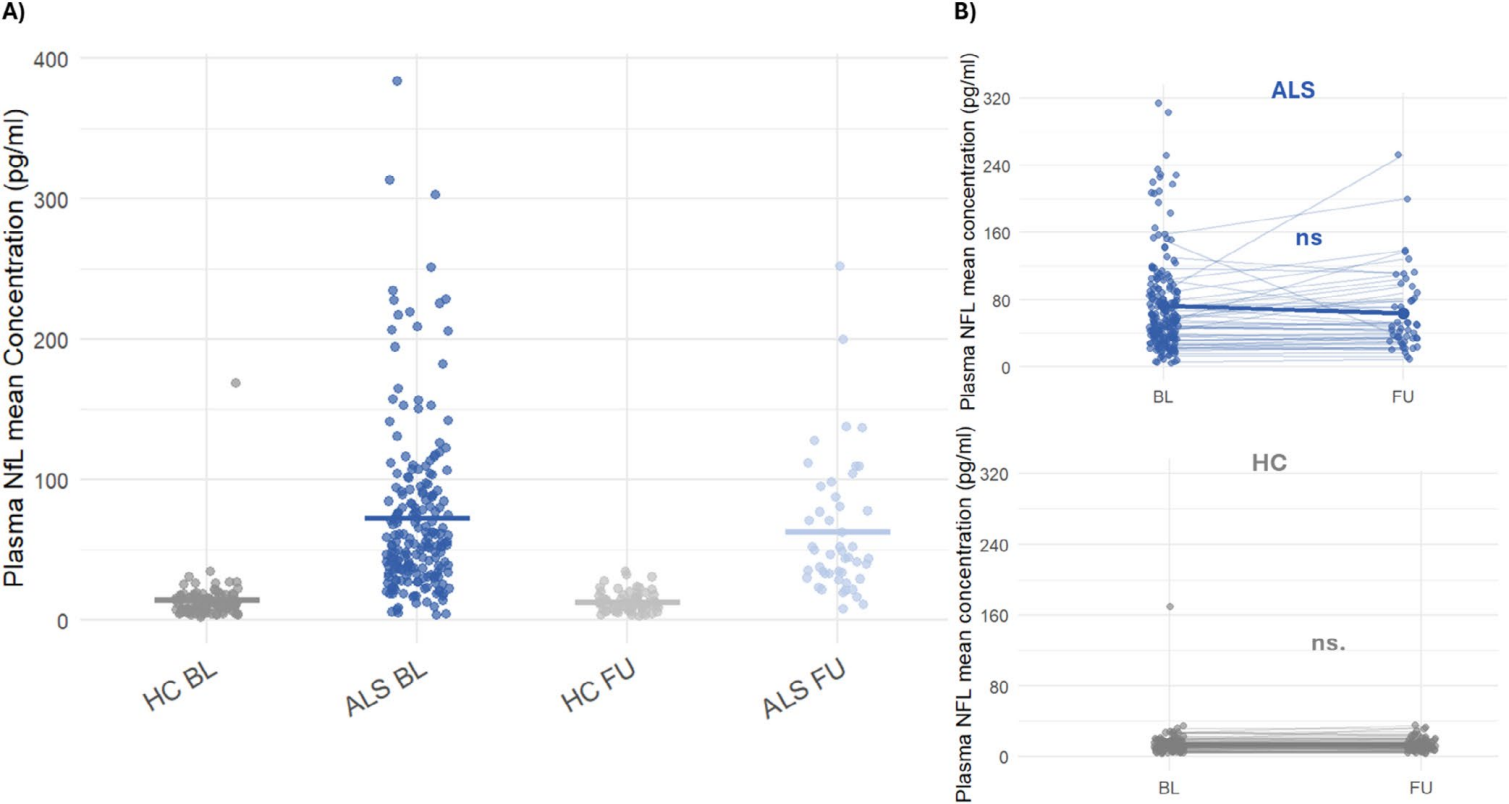
Baseline differences and longitudinal dynamics of plasma NfL concentration. (A) Group comparisons of plasma NfL concentration at baseline and follow‐up in patients and healthy controls. Points represent individual values; horizontal bars indicate group means. (B) Longitudinal trajectories of plasma NfL concentration in patients (top) and healthy controls (bottom). Lines connect repeated measures for individuals with data at both time points; points represent all available observations. BL, baseline; HC, healthy controls; FU, follow‐up (dark blue/dark gray = BL; light blue/light gray = FU). Significance levels from linear mixed‐effects models adjusted for age: *p < 0.05, **p < 0.01, ***p < 0.001. Linear mixed‐effects models were fitted using all available data, whereas longitudinal trajectories include only individuals with measurements at both time points.

Cross‐sectional analyses revealed significantly higher plasma concentrations of both p‐tau181 and NfL in people with ALS (plwALS) compared to healthy controls (HC) (Table 1, Figures 1A and 2A and Table S1). No significant group differences were observed for CSF p‐tau181. Both plasma and CSF p‐tau181 correlated positively with age at baseline (*β* = 0.04, *p* = 0.001; *β* = 0.59, *p* < 0.0001). No significant differences were observed between ALS gene mutation carriers (e.g., FUS, SOD1) and non‐carriers across any measured parameters (all *p* > 0.05). Within the ALS group, regarding APOE genotype, ε4 carriers exhibited significantly higher Aβ42/p‐tau181 and Aβ42/40 ratios compared with non‐carriers (*W* = 1581, *p* < 0.0001; *W* = 1459, *p* = 0.002, respectively). However, p‐tau181 levels did not differ between the groups in either CSF or plasma (all *p* > 0.05). When considering ATN status as combining score of amyloid and p‐tau, i.e., the presence of Alzheimer's pathology in CSF, significant differences were observed between low‐risk patients (ATN = 0) and high‐risk patients (ATN > 0) for all CSF parameters, including p‐tau181, total tau, Aβ40/42, and Aβ42/p‐tau181 ratio (all *p* < 0.001). Importantly, no differences were observed for plasma tau (*W* = 349, *p* = 0.6739).

**TABLE 1:** Demographical/clinical characteristics and values of key biomarkers of HC and ALS at baseline and follow‐up after 1 year.

	HC		ALS	
	Baseline (BL)	Follow‐up	Baseline	Follow‐up
*N* (f/m)	94 (54/40)	73 (38/45)	202 (91/111)	50
Age in years	61.0 (14.21)	63.9 (12.63)	63.5 (12.47)	61.9 (12.71)
ALSFRS‐R (M, SD)/range			35.8 (8.21)/5–48	28.1 (13.31)/4–47
Disease duration in month^a^ (M, SD)/range			34.7 (42.81)/3–287	45.9 (31.95)/9–174
Interval btw. BL and FU in month (M, SD)	14.7 (6.22)		12.4 (6.83)	
Progression rate (MD, SD)			Onset to Baseline: 0.47 (0.79)	Baseline to Follow‐up: 0.58 (1.00)
Progressor type (slow/fast) (%)			48.7/51.3	
Motor neuron involvement (UMN/LMN/equal) (%)			21.3/26.9/51.8	
King's staging (1/2/3/4/5, %)			10.0/12.7/70/7.3/0	
ALS gene mutations (*N*, %)			13, 4.6%	
ApoE genotype (*N* = 184) % (2/3, 2/4, 3/3, 3/4, 4/4)			9.8/1.1/62/23.8/3.3	
ATN^a^ (*N* = 104) % (0/1/2/3)			77.8/14.4/2.8/4.8	
Plasma p‐tau181				
*N*	94	73	202	50
p‐tau181 pg/mL mean concentration (M, SD)	1.7 (1.10)	1.87 (1.26)	3.9 (3.00)	4.6 (3.94)
Plasma neurofilament light chain (NfL)				
*N*	92	71	202	48
NfL mean concentration in pg/mL (M, SD)	14.4 (17.04)	13.3 (7.00)	73.2 (60.16)	63.5 (48.72)
Cerebrospinal fluid (CSF)				
*N*	24		104	
p‐tau181 pg/mL (M, SD)	60.3 (19.48)		48.9 (22.26)	
Total‐tau pg/mL (M, SD)	331.9 (133.20)		389.8 (204.79)	
Ratio Aß42/Aß40 (M, SD)	0.11 (0.02)		0.09 (0.024)	
Ratio Aß42/p‐tau181 (M, SD)	18.8 (5.06)		16.7 (5.99)	

*Note:* Progression rate (PR) calculated as follows: PR at baseline = (48 − ALSFRS‐R at baseline)/disease duration in month since onset, PR at Follow‐up = (ALSFRS‐R at baseline − ALSFRS‐R at follow‐up)/disease duration in month since baseline; Progressor type calculated by median split: slow = Progression rate < Median, fast = Progression rate > Median; equal = signs of upper and lower motor neuron involvement in equal matter; King's stages: 1 = symptom onset and involvement of the first neuroanatomical region (bulbar, upper, or lower limb); stage 2: involvement of a second neuroanatomical region; stage 3: involvement of a third neuroanatomical region; stage 4 = development of nutritional failure (need for gastrostomy) or respiratory failure (need for non‐invasive ventilation); stage 5: death.

Abbreviations: ALSFRS‐R, ALS Functional Rating Scale‐revised; HC, healthy controls; LMN, dominance of lower motoneuron involvement; UMN, dominance of upper motoneuron involvement; NfL, neurofilament light chain; M, mean; MD, median; SD, standard deviation.

^a^ ATN status was determined according to the ATN framework (amyloid deposition [A], tau pathology [T], and neurodegeneration [N]) using CSF Aβ42/40 ratio, p‐tau181, and total tau applying our assay‐specific cut‐off values as described in supplement 1 (0 = normal, 1 = early amyloid pathology, 2 = AD pathology, 3 = AD with neurodegeneration).

Longitudinally, neither plasma NfL nor p‐tau181 changed significantly in HC. In contrast, plasma p‐tau181 increased over time in plwALS, whereas NfL remained stable. A significant group × time interaction indicated that disease status, rather than age, was the main driver of biomarker change (Figures 1B and 2B and Table S1).

**FIGURE 3:**
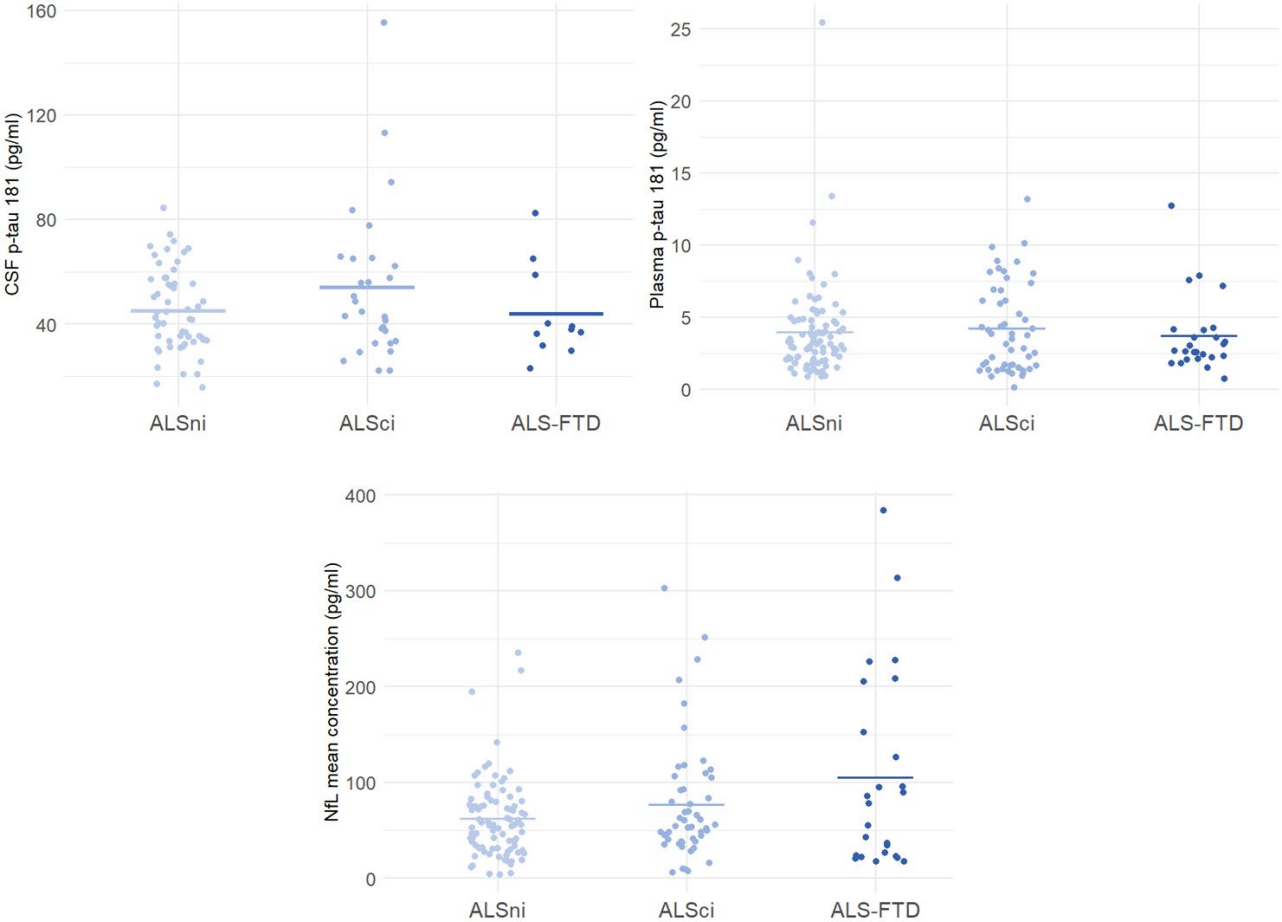
Group comparisons of biomarkers between patients' cognitive subgroups. ALSci, ALS with cognitive impairment; ALS‐FTD, ALS with additional frontotemporal dementia; ALSni, ALS without impairment.

**FIGURE 4:**
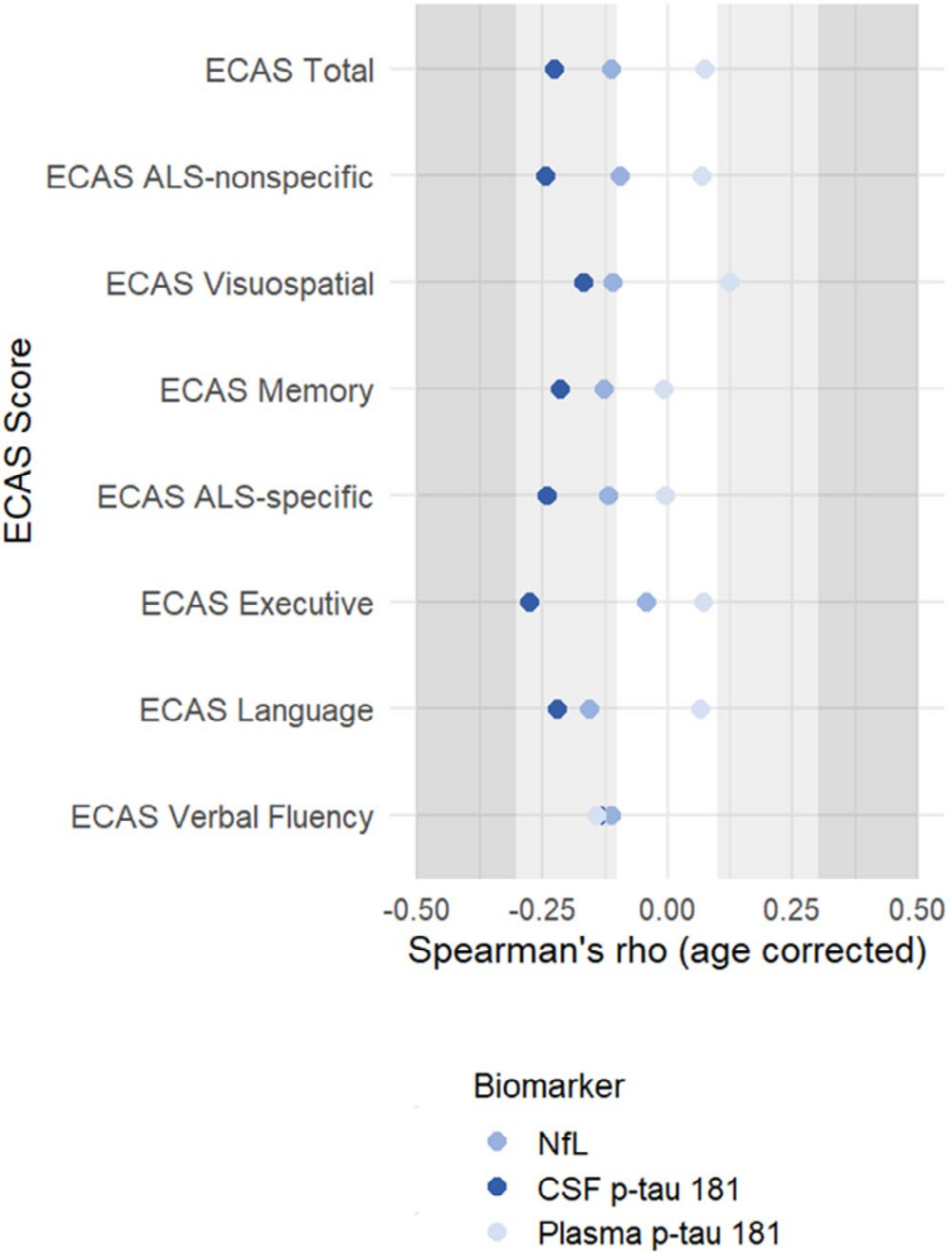
Correlations between biomarkers and ECAS Scores in ALS‐patients. ECAS, Edinburgh Cognitive and Behavioral ALS Screen; + Bonferroni‐Holm corrected for multiple comparisons; diamond = *p* < 0.4.

Regarding cognition, biomarker levels at baseline did not differ significantly among cognitively unimpaired, cognitively impaired, and ALS‐FTD subgroups (Figure 3; Table S2). Partial correlations between cognitive test performance and biomarker levels did not reach statistical significance (Figure 4; Table S3) neither for ALS‐specific domains such as verbal fluency and executive function (Spearman's *ρ* = 0.00, corrected *p* = 1.00) nor ALS‐non‐specific domains such as memory (*ρ* = 0.07, corrected *p* = 1.00). Nevertheless, numerically stronger associations were observed for CSF p‐tau181 compared with plasma p‐tau181 and NfL, suggesting a possible—but statistically non‐significant—tendency toward closer coupling between central p‐tau181 and cognitive performance (all uncorrected *p* > 0.4).

**FIGURE 5:**
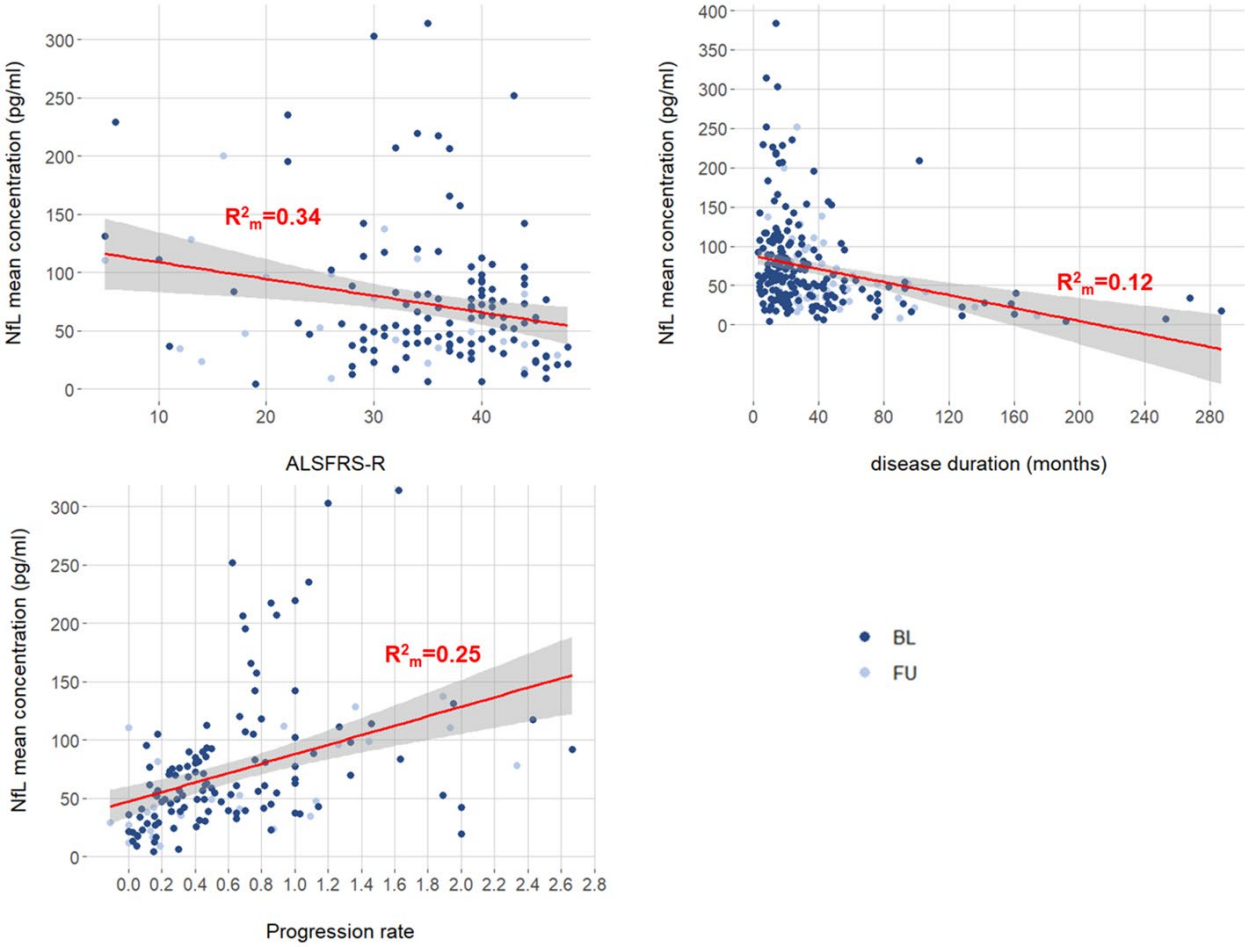
Associations between biomarkers and clinical characteristics at baseline and over time. ALSFRS‐R, ALS‐Functional Rating Scale‐Revised; Progression rate (PR) calculated as follows: PR at baseline = (48 − ALSFRS‐R at baseline)/disease duration in month since onset, PR at follow‐up = (ALSFRS‐R at baseline − ALSFRS‐R at follow‐up)/disease duration in month since baseline; *R* ^2^_m_ = marginal explained variance via fixed effects based on Mixed Linear Models; dark blue = Baseline; light blue = Follow‐up.

Analyses of biomarker associations with clinical characteristics (Figure 5, Table S4) revealed significant effects only for NfL. Higher NfL was associated with progressed disease severity (*β* = −0.05, *p* = 0.015), faster progression (*β* = 0.01, *p* < 0.001), and shorter disease duration. No sex or age interactions were detected (all *p* > 0.05; Table S4). NfL, but not p‐tau181, differed significantly between slow and fast progressors (*W* = 826, *p* < 0.0001). Taking baseline King's stages into account, significant between‐stage differences were found in NfL levels (*W* = 11.87, *p* = 0.008), ALS‐FRS‐R (*W* = 19.67, *p* < 0.001), and progression rate (*W* = 12.25, *p* = 0.007), however, not in p‐tau181 values (*W* = 0.80, *p* = 0.850), and disease duration (*W* = 3.33, *p* = 0.344) (see Table S5). Finally, plasma p‐tau181 but not NfL was elevated in patients with predominantly lower motor neuron (LMN) versus upper motor neuron (UMN) involvement (Dunn's test = 3.20, *p* = 0.004; Table S6). A significant positive correlation was observed between plasma p‐tau181 and NfL (*ρ* = 0.43, *p* < 0.0001), but not with p‐tau181 from CSF.

## 4 Discussion

In our study, plasma p‐tau181 levels were increased in plwALS, but showed no association with cognitive performance or concomitant FTD. This absence of correlation applied to both ALS‐specific and Alzheimer's disease (AD)‐related cognitive domains (e.g., memory, visuospatial skills), supporting the notion that plasma p‐tau181 in ALS does not reflect AD‐related co‐pathology in contrast to phosphorylated tau from CSF which appears to be directly related to cognitive dysfunction in AD [17]. The independence of plasma from CSF p‐tau181 further strengthens the hypothesis of a predominantly peripheral rather than central origin of plasma p‐tau181. Although the extent and pattern of cognitive impairments in our cohort were consistent with the literature, no differences in CSF‐biomarker levels emerged between the cognitive patient groups. This might reflect that the Strong criteria emphasize ALS‐specific cognitive domains, less typical for cognitive pattern in AD. As expected, ALS patients with pathological AD neurodegeneration marker levels (high‐risk ATN status) and high‐risk APOE genotype carriers showed increased CSF AD marker levels. However, p‐tau181 plasma levels remained unchanged, supporting a peripheral rather than central origin of the plasma signal. In contrast to the APOE genotype, ALS‐associated genes appear to have no specific effect, with results that are independent of monogenic and sporadic forms of the disease.

The validity of our findings is reinforced by the concordance of our results on motor impairment and other clinical markers with previous reports [2, 3, 4, 18, 19]. Plasma p‐tau181 concentrations were most markedly elevated in patients exhibiting predominant lower motor neuron (LMN) involvement, whereas NfL levels remained strongly associated with disease severity and progression but were independent of predominant UMN vs. LMN involvement, consistent with its well‐established diagnostic and prognostic utility in ALS. However, p181 could be considered to have greater diagnostic than prognostic value.

Prior studies have reported only weak correlations between plasma p‐tau181 and clinical severity measures [2, 4]. In contrast, a recent investigation employing the NULISA assay by Thomas et al. [19] demonstrated a robust association between them. Furthermore, in their study, plasma p‐tau181 correlated with ALSFRS‐R scores and survival, with higher concentrations predicting poorer clinical outcomes. This study, however, was conducted in a single‐centre pilot cohort comprising a limited sample size and utilized the novel NULISA technology, factors that may account for the observed differences to our findings. Single other studies have likewise proposed plasma p‐tau181 as a prognostic indicator of survival and the rate of disease progression [4], however without a consistent overall pattern. Therefore, the correlation to severity needs further investigations. Furthermore, p‐tau181 did not correlate with King's stages, highlighting its ineffectiveness as a marker of severity. Unfortunately, survival data were not available, which represents a limitation of our study The fact that our results converge with those from independent cohorts supports the robustness and external validity of our data in capturing the biological and clinical heterogeneity inherent to ALS. Longitudinally, plasma p‐tau181 levels increased over 1 year, while NfL remained stable. Although this trajectory suggests potential responsiveness to peripheral neurodegenerative processes, the absence of a link with disease progression argues against p‐tau181 as a prognostic biomarker in ALS. However, it may represent a valuable additional biomarker of therapy response in clinical trials. For future studies, it would be worthwhile to include additional plasma biomarkers, such as p‐tau217, which has superseded p‐tau181 as a marker of AD pathology [20] and shows high potential for distinguishing AD from non‐AD pathology [21].

In conclusion, plasma p‐tau181 is elevated in ALS but unrelated to cognitive impairment, supporting a peripheral rather than cerebral source. Its consistent association with LMN involvement and lack of correlation with disease severity and progression suggest a complementary, yet distinct, biomarker role alongside NfL.

## Author Contributions

E.K., A.H., O.P., Jos.P., G.C.P., E.D., Em.D., M.S., A.S., F.B., M.W. and M.C.S. contributed to the conception and design of the study; E.K., A.L., O.P., Jos.P., E.J.S., G.C.P., I.V., P.W., S.B., E.D., B.F., R.G., Em.D., W.G., M.S., L.B., A.S., M.W., F.B., M.C.S., N.N., Anj.S., S.T., Joh.P., and A.H. contributed to the acquisition and analysis of data; E.K. and A.H. contributed to drafting the text or preparing the figures.

## Funding

The authors have nothing to report.

## Ethics Statement

The study was conducted according to the Declaration of Helsinki and approved by the local medical ethics committees (311/14‐313/14; A2014‐0162; A2023‐0162).

## Conflicts of Interest

S.T. served on advisory boards of Lilly, Eisai, and Biogen, and was member of the independent data safety and monitoring board of the ENVISION study (Biogen). B.F. has received advisory and consultancy honoraria from AbbVie, Bial, and is an investigator on studies funded by AbbVie, Bial, Biogen, CHDI, Denali, DFG, MJFF, Takeda, Teva, UCB.

## Supporting information


**Data S1:** Methods.

**Table S1:** Results of group comparisons between HC and ALS to baseline and follow‐up.
**Table S2:** Group comparisons of biomarkers between patient's cognitive subgroups.
**Table S3:** Partial correlations between biomarkers and ECAS Scores in ALS‐patients controlling for age.
**Table S4:** Associations between biomarkers and clinical parameters dependent of timepoint.
**Table S5:** Group comparisons between different King's stages in ALS patients at baseline.
**Table S6:** Group comparisons between different ALS progressor types and motoneuron involvement at baseline.

## Data Availability

Anonymized data and scripts used for the current study are available from the corresponding author upon reasonable request.

## References

[1] C. R. Jack, Jr. , D. A. Bennett , K. Blennow , et al., “NIA‐AA Research Framework: Toward a Biological Definition of Alzheimer's Disease,” Alzheimer's & Dementia 14, no. 4 (2018): 535–562. 10.1016/j.jalz.2018.02.018 PMC5958625 29653606

[2] S. Abu‐Rumeileh , L. Scholle , A. Mensch , et al., “Phosphorylated Tau 181 and 217 Are Elevated in Serum and Muscle of Patients With Amyotrophic Lateral Sclerosis,” Nature Communications 16, no. 1 (2025): 2019. 10.1038/s41467-025-57144-7 PMC11882981 40044663

[3] V. Vacchiano , A. Mastrangelo , C. Zenesini , et al., “Elevated Plasma p‐tau181 Levels Unrelated to Alzheimer's Disease Pathology in Amyotrophic Lateral Sclerosis,” Journal of Neurology, Neurosurgery & Psychiatry 94, no. 6 (2023): 428–435. 37012065 10.1136/jnnp-2022-330709

[4] K. A. Cousins , L. M. Shaw , S. Shellikeri , et al., “Elevated Plasma Phosphorylated Tau 181 in Amyotrophic Lateral Sclerosis,” Annals of Neurology 92, no. 5 (2022): 807–818. 35877814 10.1002/ana.26462 PMC9588516

[5] M. Benatar , L. W. Ostrow , J. W. Lewcock , et al., “Biomarker Qualification for Neurofilament Light Chain in Amyotrophic Lateral Sclerosis: Theory and Practice,” Annals of Neurology 95, no. 2 (2024): 211–216. 38110839 10.1002/ana.26860 PMC10842825

[6] S. Benbrika , B. Desgranges , F. Eustache , and F. Viader , “Cognitive, Emotional and Psychological Manifestations in Amyotrophic Lateral Sclerosis at Baseline and Overtime: A Review,” Frontiers in Neuroscience 13 (2019): 951. 31551700 10.3389/fnins.2019.00951 PMC6746914

[7] J. Raaphorst , M. van Tol , M. De Visser , et al., “Prose Memory Impairment in Amyotrophic Lateral Sclerosis Patients Is Related to Hippocampus Volume,” European Journal of Neurology 22, no. 3 (2015): 547–554. 25557180 10.1111/ene.12615

[8] J. M. Cedarbaum , N. Stambler , E. Malta , et al., “The ALSFRS‐R: A Revised ALS Functional Rating Scale That Incorporates Assessments of Respiratory Function,” Journal of the Neurological Sciences 169, no. 1–2 (1999): 13–21. 10540002 10.1016/s0022-510x(99)00210-5

[9] B. R. Brooks , R. G. Miller , M. Swash , and T. L. Munsat , “El Escorial Revisited: Revised Criteria for the Diagnosis of Amyotrophic Lateral Sclerosis,” Amyotrophic Lateral Sclerosis and Other Motor Neuron Disorders 1, no. 5 (2000): 293–299. 11464847 10.1080/146608200300079536

[10] R. Balendra , A. Al Khleifat , T. Fang , and A. Al‐Chalabi , “A Standard Operating Procedure for King's ALS Clinical Staging,” Amyotrophic Lateral Sclerosis and Frontotemporal Degeneration 20, no. 3–4 (2019): 159–164. 30773950 10.1080/21678421.2018.1556696 PMC6558284

[11] D. Oender , J. Faber , C. Wilke , et al., “Evolution of Clinical Outcome Measures and Biomarkers in Sporadic Adult‐Onset Degenerative Ataxia,” Movement Disorders 38, no. 4 (2023): 654–664. 36695111 10.1002/mds.29324

[12] S. Abrahams , J. Newton , E. Niven , J. Foley , and T. H. Bak , “Screening for Cognition and Behaviour Changes in ALS,” Amyotrophic Lateral Sclerosis and Frontotemporal Degeneration 15, no. 1–2 (2014): 9–14. 23781974 10.3109/21678421.2013.805784

[13] M. Loose , C. Burkhardt , H. Aho‐Özhan , et al., “Age and Education‐Matched Cut‐Off Scores for the Revised German/Swiss‐German Version of ECAS,” Amyotrophic Lateral Sclerosis and Frontotemporal Degeneration 17, no. 5–6 (2016): 374–376. 27027323 10.3109/21678421.2016.1162814

[14] M. J. Strong , S. Abrahams , L. H. Goldstein , et al., “Amyotrophic Lateral Sclerosis‐Frontotemporal Spectrum Disorder (ALS‐FTSD): Revised Diagnostic Criteria,” Amyotrophic Lateral Sclerosis and Frontotemporal Degeneration 18, no. 3–4 (2017): 153–174. 28054827 10.1080/21678421.2016.1267768 PMC7409990

[15] K. Rascovsky , J. R. Hodges , D. Knopman , et al., “Sensitivity of Revised Diagnostic Criteria for the Behavioural Variant of Frontotemporal Dementia,” Brain 134, no. 9 (2011): 2456–2477. 21810890 10.1093/brain/awr179 PMC3170532

[16] M. L. Gorno‐Tempini , A. E. Hillis , S. Weintraub , et al., “Classification of Primary Progressive Aphasia and Its Variants,” Neurology 76, no. 11 (2011): 1006–1014. 21325651 10.1212/WNL.0b013e31821103e6 PMC3059138

[17] R. L. Hamilton and R. Bowser , “Alzheimer Disease Pathology in Amyotrophic Lateral Sclerosis,” Acta Neuropathologica 107 (2004): 515–522. 15024584 10.1007/s00401-004-0843-1

[18] Y. Kojima , T. Kasai , Y.‐i. Noto , et al., “Amyotrophic Lateral Sclerosis: Correlations Between Fluid Biomarkers of NfL, TDP‐43, and Tau, and Clinical Characteristics,” PLoS One 16, no. 11 (2021): e0260323. 34843548 10.1371/journal.pone.0260323 PMC8629269

[19] E. V. Thomas , C. Han , W. J. Kim , et al., “ALS Plasma Biomarkers Reveal Neurofilament and pTau Correlate With Disease Onset and Progression,” Annals of Clinical and Translational Neurology 12, no. 4 (2025): 714–723. 39913612 10.1002/acn3.70001 PMC12040516

[20] K. K. Petersen , M. Milà‐Alomà , Y. Li , et al., “Predicting Onset of Symptomatic Alzheimer's Disease With Plasma p‐tau217 Clocks,” Nature Medicine 32 (2026): 1085–1094. 10.1038/s41591-026-04206-y PMC13004683 41714746

[21] N. J. Ashton , W. S. Brum , G. Di Molfetta , et al., “Diagnostic Accuracy of a Plasma Phosphorylated Tau 217 Immunoassay for Alzheimer Disease Pathology,” JAMA Neurology 81, no. 3 (2024): 255–263. 38252443 10.1001/jamaneurol.2023.5319 PMC10804282

