# Supplementary material for: Impact of Plasma p‐tau181 on Cognition, Motor Phenotypes, and Disease Course in ALS
Source: Ann Clin Transl Neurol. 2026 May 4:10.1002/acn3.70423. Online ahead of print. doi: 10.1002/acn3.70423 (PMC13395029; doi:10.1002/acn3.70423)
Supplement: Supplementary file 1 — Data S1: Methods. [file ACN3-9999-0-s002.docx]

**SUPPLEMENT 1 - Methods -**

*Participants:*

The DZNE Clinical Registry Study of Neurodegenerative Diseases (DESCRIBE) cohort is a multicenter, prospective, longitudinal observational study conducted by the German Center for Neurodegenerative Diseases (DZNE). The multicenter, longitudinal Degeneration Controls and Relatives cohort (DANCER) serves to recruit healthy controls. We analyzed 202 plwALS or ALS-FTD (from DESCRIBE-ALS/FTD) as well as 94 healthy control persons (from DANCER). All patients were characterized by ALS Functional Rating Scale-Revised (ALSFRS-R) ^1^ and diagnosed according to the revised El Escorial Criteria ^2^. Furthermore, were categorized according to the King stages^3^. These include the following categories: 1=symptom onset and involvement of the first neuroanatomical region (bulbar, upper, or lower limb); stage 2: involvement of a second neuroanatomical region; stage 3: involvement of a third neuroanatomical region; stage 4=development of nutritional failure (need for gastrostomy) or respiratory failure (need for non-invasive ventilation); stage 5: death.

Additionally, all patients were categorized according to the King stages.Additionally, 117 age- and education-matched healthy controls from Rostock University Medical Center, Germany, were recruited via public advertising to serve as a normative group for the cognitive categorization of ALS patients. These participants were screened using the Montreal Cognitive Assessment (MoCA) ^4^ and persons with a score below 26 were excluded. Overall, participants with a history of brain injury, epilepsy, or psychiatric illness were excluded as well.

*Measurement and analyses of Biomarkers:*

*CSF markers*:

the neurodegeneration panel from CSF included amyloid-β (Aβ38 pg/ml, Aβ40 pg/ml, Aβ42 pg/ml), total tau protein (total-tau pg/ml), phosphorylated tau (p-tau181 pg/ml), the Aβ42/40 ratio, and the Aβ42/p-tau-181 ratio, where higher values in tau scores and lower ratios indicates increased risk for AD. Pathological thresholds were derived from Gaussian mixture models procedure (73.65 pg/ml for p-tau, 510.9 for total-tau, 0.08 for ratio Aβ42/40, and 9.24 for ratio Aβ42/p-tau181). Notably, the current cut-offs should be considered as specific for DZNE research cohorts. The manual method description as well as the principle of cut-off value determination is available from previous publications of DZNE ^5, 6^.

*Plasma p-tau181:*

Blood plasma measures of p-Tau-181 were conducted using the Quanterix® SIMOA® Assay (104618, Quanterix® Corp., Billerica, USA) according to the manufacturer´s instructions, with samples run in technical duplicates and a maximum accepted coefficient of variance of 20%. Additional to manufacturer´s kit controls, an internal aliquoted plasma samples served as inter-run control.

*Plasma NfL:*

NfL mean concentrations were determined using the SIMOA NF-light Advantage kit on a HDI analyzer Quanterix by a blinded experimenter according to the manufacturer’s instructions as previously described ^7^.

*Neuropsychological measurement*

The neuropsychological assessment included the German version of the “Edinburgh Cognitive and Behavioural ALS Screen” (ECAS) ^8, 9^. The ECAS comprises 15 subtests assessing five cognitive domains: ALS specific functions (verbal fluency, executive functions including social cognition, and language) and ALS-non-specific functions (memory and visuospatial abilities). We considered sub scores for each cognitive subdomain, as well as the ALS-specific score, the ALS-nonspecific score, and the ECAS total score. Cognitive impairments were classified according to the revised Strong criteria ^10^. Based on this classification, all individuals with ALS without FTD were categorized as either “non-impaired” (ALSni) or “cognitively impaired” (ALSci). To determine cognitive impairment, z-scores were calculated for all ECAS-domains and total score, using the locally acquired age- and education matched healthy control sample. A z-score ≤ -2 was considered as indicative of impairment. Patients with ALS and FTD were categorized according Raskovsky et al. 2009 respectively Gorno-Tempini et al., 2011 criteria.

*Statistical analyses*

For cross-sectional group comparisons, linear mixed models included diagnostic group (ALS vs. HC), the timepoint and age at visit as fixed effects, with a random intercept for each participant to account for inter-individual variability. For longitudinal analysis, data from two time points (Baseline and Follow-up) were included, and the model incorporated time (visit), group, and their interaction as fixed effects, again controlling for age and including random intercepts per participant. This modelling approach allowed for a unified framework to assess both between-group differences and within-subject changes over time. Estimated marginal means (EMMs) and post-hoc contrasts (Tukey-adjusted) were computed. All analyses were conducted in R (version 4.4.3). For group comparisons between cognitive subgroups, King`s stages as well as motoneuron involvement at baseline, Kruskal Wallis Tests with Dunn tests for post hoc comparisons were conducted while nominal data were calculated by Pearson's Chi-squared test. The group comparison between slow and fast progression rate was performed with Mann Whitney U-Test. Partial correlations were conducted to assess age corrected associations between key biomarkers and cognitive scores.

1. Cedarbaum JM, Stambler N, Malta E, et al. The ALSFRS-R: a revised ALS functional rating scale that incorporates assessments of respiratory function. Journal of the neurological sciences. 1999;169(1-2):13–21.

2. Brooks BR, Miller RG, Swash M, Munsat TL. El Escorial revisited: revised criteria for the diagnosis of amyotrophic lateral sclerosis. Amyotrophic lateral sclerosis and other motor neuron disorders. 2000;1(5):293–9.

3. Balendra R, Al Khleifat A, Fang T, Al-Chalabi A. A standard operating procedure for King’s ALS clinical staging. Amyotrophic Lateral Sclerosis and Frontotemporal Degeneration. 2019;20(3-4):159–64.

4. Nasreddine ZS, Phillips NA, Bédirian V, et al. Montreal cognitive assessment. The American Journal of Geriatric Psychiatry. 2003.

5. Jessen F, Spottke A, Boecker H, et al. Design and first baseline data of the DZNE multicenter observational study on predementia Alzheimer’s disease (DELCODE). Alzheimer's research & therapy. 2018;10:1–10.

6. Jessen F, Wolfsgruber S, Kleineindam L, et al. Subjective cognitive decline and stage 2 of Alzheimer disease in patients from memory centers. Alzheimer's & dementia. 2023;19(2):487–97.

7. Oender D, Faber J, Wilke C, et al. Evolution of clinical outcome measures and biomarkers in sporadic adult‐onset degenerative ataxia. Movement disorders. 2023;38(4):654–64.

8. Abrahams S, Newton J, Niven E, Foley J, Bak TH. Screening for cognition and behaviour changes in ALS. Amyotrophic lateral sclerosis and frontotemporal degeneration. 2014;15(1-2):9–14.

9. Loose M, Burkhardt C, Aho-Özhan H, et al. Age and education-matched cut-off scores for the revised German/Swiss-German version of ECAS. Amyotrophic Lateral Sclerosis and Frontotemporal Degeneration. 2016;17(5-6):374–6.

10. Strong MJ, Abrahams S, Goldstein LH, et al. Amyotrophic lateral sclerosis-frontotemporal spectrum disorder (ALS-FTSD): Revised diagnostic criteria. Amyotrophic lateral sclerosis and frontotemporal degeneration. 2017;18(3-4):153–74.
