# Supplementary material for: Impact of Plasma p‐tau181 on Cognition, Motor Phenotypes, and Disease Course in ALS
Source: Ann Clin Transl Neurol. 2026 May 4:10.1002/acn3.70423. Online ahead of print. doi: 10.1002/acn3.70423 (PMC13395029; doi:10.1002/acn3.70423)
Supplement: Supplementary file 2 — Table S1: Results of group comparisons between HC and ALS to baseline and follow‐up. Table S2: Group comparisons of biomarkers between patient's cognitive subgroups. Table S3: Partial correlations between biomarkers and ECAS Scores in ALS‐patients controlling for age. Table S4: Associations between biomarkers and clinical parameters dependent of timepoint. Table S5: Group comparisons between different King's stages in ALS patients at baseline. Table S6: Group comparisons between different ALS progressor types and motoneuron involvement at baseline. [file ACN3-9999-0-s001.docx]

**Supplement 2 - Results -**

Table S1. Results of group comparisons between HC and ALS to Baseline and Follow up

|  | | **ß***^+^* | **SE** | **T (df)** | **p** |
| --- | --- | --- | --- | --- | --- |
| Plasma p-tau181 | *Fixed Effects* | | | | |
|  | BL vs. FU | 0.04 | 0.265 | 0.15 | 0.885 |
|  | HC vs. ALS | 2.15 | 0.317 | 6.78 | **<0.0001***** |
|  | Age | 0.04 | 0.011 | 3.56 | **0.001***** |
|  | BL vs. FU * HC vs. ALS | 1.00 | 0.387 | 2.59 | **0.011*** |
|  | *Post-hoc comparisons* | | | | |
|  | BL-HC vs. BL-ALS | 2.15 | 0.317 | -6.78 | **<0.0001***** |
|  | FU-HC vs. FU-ALS | -3.15 | 0.412 | -7.65 | **<0.0001***** |
|  | BL-HC vs. FU-HC | -0.04 | 0.265 | -0.15 | 0.999 |
|  | BL-ALS vs. FU-ALS | 1.04 | 0.284 | -3.67 | **0.002**** |
| CSF p-tau181^++^ | *Fixed effects* | | | | |
|  | HC vs. ALS | -5.00 | 3.714 | -1.35 | 0.180 |
|  | Age | 0.59 | 0.118 | 5.03 | **<0.0001***** |
|  | *Post-hoc comparisons* | | | | |
|  | BL-HC vs. BL-ALS | 5.00 | 3.710 | 1.35 | 0.180 |
| NfL | *Fixed effects* | | | | |
|  | BL vs. FU | 0.77 | 3.101 | -0.25 | 0.803 |
|  | HC vs. ALS | 57.88 | 5.883 | 9.84 | **<0.0001***** |
|  | Age | 0.57 | 0.212 | 2.68 | **0.008**** |
|  | BL vs. FU * HC vs. ALS | 6.31 | 4.625 | 1.37 | 0.175 |
|  | *Post-hoc comparisons* | | | | |
|  | BL-HC vs. BL-ALS | -57.88 | 5.880 | -9.84 | **<0.0001***** |
|  | FU-HC vs. FU-ALS | -64.19 | 6.760 | -9.49 | **<0.0001***** |
|  | BL-HC vs. FU-HC | 0.77 | 3.100 | 0.25 | 0.995 |
|  | BL-ALS vs. FU-ALS | -5.54 | 3.450 | 1.61 | 0.379 |

HC=Healthy Controls; +: linear mixed models with fixed effects of factors diagnosis and timepoint,

interaction effects between them, covariate age, as well as post hoc single group comparisons;

++: only baseline data available

Table S2. Group comparisons of biomarkers between patient’s cognitive subgroups

|  | **ALSni** | **ALSci** | **ALS-FTD** | **Group comparisons [Chi^2^(adj. p)]^+^** | | |
| --- | --- | --- | --- | --- | --- | --- |
|  |  |  |  | ALSci vs. ALSni | ALSni vs. ALS-FTD | ALSci vs. ALS-FTD |
| N (f/m) | 89 (41/48) | 52 (21/31) | 25 (9/16) | Chi^2^=0.98. p=0.611^++^ | | |
| Age in years | 61.7 (12.28) | 65.8 (10.37) | 70.6 (8.99) | 1.67 (0.094) | **3.51 (0.001)***** | 2.06 (0.079) |
| ALSFRS-R (M. SD) | 36.8 (7.40) | 34.1 (8.71) | 38.3 (7.27) | -1.76 (0.234) | 0.27 (0.786) | 1.14 (0.507) |
| Disease duration  in month (M, SD)/ range | 32.2 (40.40) /  3-268 | 35.0 (46.55) / 4-253 | 47.3 (57.82) / 8-287) | -0.738 (0.461) | 1.68 (0.186) | 2.09 (0.109) |
| Progression rate (MD, SD) | 0.45 (0.47) | 0.64 (1.20) | 0.36 (0.60) | -1.53 (0.376) | 0.46 (0.644) | -1.23 (0.437) |
| APOE genotype %  (2/3, 2/4, 3/3,3/4,4/4) | 8.2/1.2/60.5/26.8/2.3 | 12.8/0/61.7/23.4/2.1 | 15/0/60/10/15 |  |  |  |
| ATN status ^1^ %  (0/1/2/3) | 84/12/2/2 | 81.4/14.8/0/3.8 | 63.6/18.2/9.1/9.1 |  |  |  |
| ALS gene mutations (N) | 3 | 4 | 3 |  |  |  |
| **Plasma** |  |  |  |  |  |  |
| N | 74 | 37 | 8 |  |  |  |
| P-tau181 pg/ml (M, SD) | 4.00 (3.20) | 4.21 (3.07) | 3.69 (2.61) | -0.03 (0.974) | -0.47 (1) | -0.41 (1) |
| NfL (pg/ml) | 62.4 (41.76) | 76.9 (62.58) | 105.5 (101.63) | 0.90 (0.862) | 1.06 (0.862) | 0.34 (0.862) |
| **CSF** | | | | | | |
| N | 25 | 27 | 9 |  | | |
| P-tau181 pg/ml (M, SD) | 46.8 (15.50) | 53.93 (29.73) | 46.5 (18.10) | 0.46 (1) | -0.33 (1) | -0.59 (1) |
| **Cognition (ECAS)** | | | | | | |
| N | 74 | 37 | 8 |  | | |
| ECAS Language (z)^+++^ | 0.3 (0.73) | -0.8 (1.48) | -0.7 (0.88) | **-4.15 (0.000)***** | **-3.02 (0.005)**** | -0.74 (0.459) |
| ECAS Verbal fluency (z) | -0.3 (0.89) | -1.5 (1.07) | -1.8 (1.30) | **-5.23 (0.000)***** | **-3.23 (0.003)**** | -0.38 (0.701) |
| ECAS Executive (z) | 0.0 (0.91) | -2.2 (2.00) | -2.7 (2.11) | **-5.87 (0.000)***** | **-3.61 (0.001)***** | -0.42 (0.676) |
| ECAS ALS-specific (z) | -0.1 (0.88) | -2.3 (1.26) | -2.6 (1.95) | **-7.28 (0.000)***** | **-3.99 (0.001)***** | -0.05 (0.962) |
| ECAS Memory (z) | 0.1 (1.02) | -1.0 (1.40) | -2.1 (1.79) | **-4.34 (0.000)***** | **-3.78 (0.000)***** | -1.37 (0.170) |
| ECAS Visual-spatial (z) | -0.6 (2.21) | -1.8 (3.45) | -4.4 (7.29) | -1.96 (0.111) | -2.08 (0.111) | -0.98 (0.330) |
| ECAS nonspecific (z) | 0.0 (1.05) | -1.2 (1.49) | -2.4 (2.23) | **-4.27 (0.000)***** | **-3.70 (0.000)***** | -1.33 (0.184) |
| ECAS total score (z) | -0.1 (0.94) | -2.3 (1.42) | -3.1 (2.14) | **-6.99 (0.000)***** | **-4.18 (0.000)***** | -0.39 (1) |

ALSni=ALS without impairment; ALSci=ALS with cognitive impairment; ALS-FTD=ALS with additional frontotemporal dementia;

ALSFRS-R=ALS-Functional Rating Scale-Revised; M=mean; MD=median; SD=standard deviation; ECAS=Edinburgh Cognitive and Behavioural ALS Screen; ^+++^age and education corrected Scores; ^+^Kruskal-Wallis H-statistic with Dunn tests for post-hoc comparisons, adjusted p for multiple comparisons (Bonferroni-Holm); ^++^ Pearson's Chi-squared test; *p<0.05; **p<0.01; ***p<0.001; ^1^ ATN status was determined according to the ATN framework(1) (amyloid deposition [A], tau pathology [T], and neurodegeneration [N]) using CSF Aβ42/40 ratio, p-tau181, and total tau applying our assay-specific cut-off values as described in supplement 1 (0=normal, 1=early amyloid pathology, 2=AD pathology, 3=AD with neurodegeneration)

Table S3. Partial correlations between biomarkers and ECAS Scores in ALS-patients controlling for age

| **Biomarker** | **Variable** | **spearman's rho** | **p_value** | **corrected p-value^+^** | **N** |
| --- | --- | --- | --- | --- | --- |
| Plasma NfL  mean concentration (pg/ml) | ECAS Language | -0.11 | 0.219 | 1 | 126 |
|  | ECAS Verbal fluency | -0.16 | 0.089 | 0.712 | 121 |
|  | ECAS Executive | -0.04 | 0.635 | 1 | 123 |
|  | ECAS ALS-specific | -0.12 | 0.203 | 1 | 122 |
|  | ECAS Memory | -0.13 | 0.166 | 1 | 122 |
|  | ECAS Visual-spatial | -0.11 | 0.226 | 1 | 126 |
|  | ECAS ALS-nonspecific | -0.09 | 0.307 | 1 | 120 |
|  | ECAS total score | -0.11 | 0.223 | 1 | 119 |
| Plasma p-tau181  mean concentration (pg/ml) | ECAS Language | -0.14 | 0.115 | 0.920 | 126 |
|  | ECAS Verbal fluency | 0.06 | 0.484 | 1 | 121 |
|  | ECAS Executive | 0.07 | 0.430 | 1 | 123 |
|  | ECAS ALS-specific | 0.00 | 0.959 | 1 | 122 |
|  | ECAS Memory | 0.12 | 0.165 | 1 | 126 |
|  | ECAS Visual-spatial | -0.01 | 0.941 | 1 | 122 |
|  | ECAS ALS-nonspecific | 0.07 | 0.464 | 1 | 120 |
|  | ECAS total score | 0.07 | 0.431 | 1 | 119 |
| CSF p-tau181  mean concentration (pg/ml) | ECAS Language | -0.14 | 0.278 | 0.400 | 67 |
|  | ECAS Verbal fluency | -0.22 | 0.085 | 0.400 | 64 |
|  | ECAS Executive | -0.28 | **0.027*** | 0.216 | 65 |
|  | ECAS ALS-specific | -0.24 | 0.062 | 0.378 | 63 |
|  | ECAS Memory | -0.21 | 0.096 | 0.400 | 63 |
|  | ECAS Visual-spatial | -0.17 | 0.181 | 0.400 | 66 |
|  | ECAS ALS-nonspecific | -0.24 | 0.054 | 0.378 | 64 |
|  | ECAS total score | -0.22 | 0.080 | 0.400 | 63 |

ECAS=Edinburgh Cognitive and Behvioural ALS Screen; + Bonferroni-Holm corrected for multiple comparisons; *p<0.05

Table S4. Associations between biomarkers and clinical parameters dependent of timepoint

| **Biomarker** |  | **ß** | **SE** | **t** | **p** | **Model R^2^_m_** | **Model R^2^_c_** |
| --- | --- | --- | --- | --- | --- | --- | --- |
| **Disease duration** | CSF p-tau181 (only Baseline) | -0.28 | 0.203 | -1.35 | 0.179 | 0.12 | 0.99 |
|  | Plasma p-tau181 mean concentration pg/ml | 0.45 | 0.804 | 0.56 | 0.580 |  |  |
|  | Follow-up | 9.74 | 4.454 | 2.19 | **0.040*** |  |  |
|  | NfL mean concentration (pg/ml) | -0.21 | 0.060 | -3.41 | **<0.001***** |  |  |
|  | Age | 0.86 | 0.344 | 2.51 | **0.014*** |  |  |
|  | Sex | 8.08 | 8.490 | 0.95 | 0.343 |  |  |
|  | Follow-up * Plasma p-tau181 mean concentration pg/ml | -0.02 | 0.643 | -0.03 | 0.977 |  |  |
|  | Follow-up * NFL | 0.07 | 0.059 | 1.27 | 0.215 |  |  |
| **Progression Rate** | CSF p-tau181 (only Baseline) | 0.01 | 0.005 | 1.47 | 0.148 | 0.25 | 0.76 |
|  | Plasma p-tau181 mean concentration pg/ml | 0.01 | 0.048 | 0.20 | 0.844 |  |  |
|  | Follow-up | -0.23 | 0.844 | 0.27 | 0.790 |  |  |
|  | NfL mean concentration (pg/ml) | 0.01 | 0.002 | 3.87 | **<0.001***** |  |  |
|  | Age | -0.01 | 0.011 | -0.77 | 0.446 |  |  |
|  | Sex | 0.19 | 0.243 | 0.77 | 0.447 |  |  |
|  | Follow-up * Plasma p-tau181 mean concentration pg/ml | 0.01 | 0.064 | 0.10 | 0.921 |  |  |
|  | Follow-up * NFL | 0.00 | 0.007 | 0.55 | 0.584 |  |  |
| **ALSFRS-R** | CSF p-tau181 (only Baseline) | -0.01 | 0.048 | -0.19 | 0.848 | 0.34 | 0.98 |
|  | Plasma p-tau181 mean concentration pg/ml | -0.74 | 0.447 | 1.65 | 0.104 |  |  |
|  | Follow-up | -0.81 | 5.897 | 0.14 | 0.905 |  |  |
|  | NfL mean concentration (pg/ml) | -0.05 | 0.020 | 2.50 | **0.015*** |  |  |
|  | Age | 0.04 | 0.100 | 0.42 | 0.680 |  |  |
|  | Sex | -6.89 | 2.213 | -3.11 | **0.003**** |  |  |
|  | Follow-up * Plasma p-tau181 mean concentration pg/ml | -0.23 | 0.440 | -0.54 | 0.603 |  |  |
|  | Follow-up * NfL | -0.06 | 0.050 | -1.15 | 0.384 |  |  |

ALSFRS-R=ALS-Functional Rating Scale-Revised; SE=standard estimation; Progression rate (PR) was calculated as follows: PR at baseline = (48-ALSFRS-R at baseline)/ disease duration in month since onset, PR at Follow up = (ALSFRS-R at baseline - ALSFRS-R at follow-up)/ disease duration in month since baseline; R^2^_m_ =marginal explained variance via fixed effects, R^2^_c_ = conditional explained variance including random effects

Table S5. Group comparisons between different King`s stages in ALS patients at baseline

|  | **King`s stage** | | | | **Overall comparison^+^** | **Post hoc comparisons (adj. p)^++^** | | | | | |
| --- | --- | --- | --- | --- | --- | --- | --- | --- | --- | --- | --- |
|  | **1** | **2** | **3** | **4** |  | **1 vs.2** | **1 vs. 3** | **1 vs. 4** | **2 vs. 3** | **2 vs. 4** | **3 vs. 4** |
| ALS-FRS-R  (M, SD) | 40.9  (6.87) | 39.5  (5.19) | 34.7  (7.53) | 24.8  (7.36) | **W=19.67, p<0.001** | 0.745 | **0.045*** | **<0.001***** | 0.090 | **0.001**** | **0.018**** |
| Disease duration in month (M, SD) | 30.5  (23.06) | 30.1  (15.63) | 34.4  (48.78) | 34.8  (23.18) | W=3.33, p=0.344 | p=1 | p=1 | p=1 | p=1 | p=1 | p=1 |
| Progression rate  (MD, SD) | 0.4  (0.62) | 0.3  (0.28) | 0.7  (0.95) | 0.9  (0.42) | **W=12.25, p=0.007**** | p=0.520 | p=0.061 | **p=0.013*** | p=0.232 | p=0.060 | p=0.232 |
| **Plasma** |  |  |  |  |  |  |  |  |  |  |  |
| p-tau181 pg/ml  (M, SD) | 3.3  (1.98) | 3.5  (1.74) | 3.9  (3.42) | 5.2  (4.10) | W=0.80, p=0.850 | p=1 | p=1 | p=1 | p=1 | p=1 | p=1 |
| NfL pg/ml  (M, SD) | 40.5  (44.63) | 78.2  (72.09) | 80.3  (66.32) | 102.0  (65.26) | **W=11.87, p=0.008**** | p=0.172 | **p=0.012*** | **p=0.012*** | p=0.566 | p=0.475 | p=0.475 |

ALSFRS-R=ALS-Functional Rating Scale-Revised; M=mean; SD=standard deviation; Progression rate (PR) was calculated as follows:

PR at baseline = (48-ALSFRS-R at baseline)/ disease duration in month since onset, ^+^Kruskal Wallis Test with ^++^Dunn tests for post hoc comparisons with

Bonferroni Holm correction for multiple comparisons; *p<0.05; **p<0.01; ***p<0.001

Table S6. Group comparisons between different ALS progressor types and motoneuron involvement at baseline

|  | **Progression** |  |  | **Motoneuron involvement** | |  |  |  |  |
| --- | --- | --- | --- | --- | --- | --- | --- | --- | --- |
|  | slow | fast | slow vs. fast^+^ | UMN dominant | LMN dominant | UMN=LMN  (no dominance) | UMN vs. LMN◊ | UMN vs.  no dominance◊ | LMN vs.  no dominance◊ |
|  |  |  |  |  |  |  | [Chi^2^(adj. p)]^+^ | | |
| Age in years  (M. SD) | 65.0 (10.28) | 61.6 (13.09) | W=1953, p=0.186 | 62.2 (11.59) | 64.1 (12.99) | 58.3 (13.44) | 0.70 (0.703) | 0.93 (0.703) | 1.90 (0.174) |
| ALSFRS-R  (M, SD) / range | 39.1 (6.18) /  19-48 | 32.6 (8.90) /  5-45 | **W= 2491, p<0.0001***** | 35.4 (10.82) /  6-47 | 35. (6.53) /  5-48 | 34.2 (7.97) /  11-46 | -0.74 (0.924) | 1.22 (0.667) | 0.68 (0.924) |
| Disease duration in month  (M, SD) / range | 51.0 (58.05) /  9-287 | 17.1 (9.92) /  3-56 | **W=2767.5, p<0.0001***** | 32.7 (35.65) /  4-160 | 28.8 (36.03) /  5-268 | 41.3 (54.78) /  7-287 | 0.06 (1) | -0.65 (1) | -0.73 (1) |
| Progression rate  (MD SD) | 0.3 (0.14) | 0.9 (0.92) | **W=0, p<0.0001***** | 0.5 (1.61) | 0.4 (0.45) | 0.6 (0.49) | 0.96 (0.990) | -0.97 (0.990) | -0.12 (0.990) |
|  |  |  |  |  |  |  |  |  |  |
| **Plasma** |  |  |  |  |  |  |  |  |  |
| p-tau181 pg/ml  (M. SD) | 3.5 (2.18) | 3.7 (2.65) | W=1707, p=0.989 | 2.5 (1.58) | 4.6 (3.81) | 3.5 (2.24) | **3.20 (0.004**)** | -1.68 (0.186) | 1.41 (0.186) |
| NfL (pg/ml) | 49.1 (25.70) | 103.1 (71.19) | **W=826, p<0.0001***** | 76.9 (60.35) | 79.0 (70.11) | 77.9 (65.13) | -0.12 (1) | 0.16 (1) | 0.08 (1) |
| **CSF** |  |  |  |  |  |  |  |  |  |
| p-tau181 pg/ml  (M. SD) | 54.8 (19.79) | 47.9 (26.68) | W=514, p=0.077 | 59.2 (17.09) | 54.6 (28.13) | 39.6 (15.01) | -0.97 (0.331) | 1.85 (0.192 | 1.46 (0.287) |

HC=Healthy Controls; ALSFRS-R=ALS-Functional Rating Scale-Revised; M=mean; MD=median; SD=standard deviation; Progression rate (PR) was calculated as follows: PR at baseline = (48-ALSFRS-R at baseline)/ disease duration in month since onset, PR at Follow up = (ALSFRS-R at baseline - ALSFRS-R at follow-up)/ disease duration in month since baseline; Progressor type calculated by median split: slow=Progression rate < median, fast= Progression rate > Median; UMN=dominance of upper motoneuron involvement, LMN= dominance of lower motoneuron involvement, equal=signs of upper and lower motor neuron involvement in equal matter; ^+^Mann Whitney U-Test; ^++^ Pearson's Chi-squared test; ◊Kruskal Wallis Test with Dunn tests for post hoc comparisons; *p<0.05; **p<0.01; ***p<0.001

**References:**

1. Jack Jr CR, Bennett DA, Blennow K, Carrillo MC, Dunn B, Haeberlein SB, et al. NIA‐AA research framework: toward a biological definition of Alzheimer's disease. Alzheimer's & dementia. 2018;14(4):535–62.
